# News and Miscellany

**Published:** 1903-08

**Authors:** 


### News and Miscellany.

---

WILL REPAIR YOUR ELECTRICAL MACHINES.—Static and all electrical medical apparatus put in running order. I am also agent for electrical and X-ray apparatus. Oliver Brush, 710 Colorado Street, Austin, Texas.

---

NEW ORLEANS POLYCLINIC.—Seventeenth annual session opens November 2, 1903, and closes May 28, 1904. Physicians will find the Polyclinic an excellent means for posting themselves upon modern progress in all branches of medicine and surgery. The specialties are fully taught, including laboratory work. For further information, address New Orleans Polyclinic, postoffice box 797, New Orleans, La.

---

THE I. & G. N. ILLUSTRATOR AND NARRATOR—Diversified Farming Along the Line of the International and Great Northern Railroad—"The Texas Road." We have received a copy of this splendid work. It is beautifully illustrated with scenes, showing the various farming industries of the State and containing a wealth of information as to Texas industries, invaluable to those who think of seeking homes in this land flowing with milk and honey and coal oil and things. Mr. D. J. Price, G. P. and T. A. of the road, Palestine, Texas; likewise the editor and getter-up of this valuable publication, will send a copy to any address on receipt of a 2-cent stamp. Mention this notice.

---

STERILIZED SLEEPERS AND DAY COACHES.—Dr. W. G. Jameson, Chief Surgeon of the I. & G. N. R. R., stated at the recent meeting of railroad surgeons at the office of State Health Officer Tabor, that the I. & G. N. system is disinfecting all cars by means of the Germicide Gas Generator (formaldehyde machine). This is the only road in the United States where practical disinfection of all cars is carried out; the assumption being that all sleepers are infected at least with the germs of tuberculosis all the time.

Gen. P. J. A. Cleary.—Dr. Cleary, Chief Surgeon, U. S. A., Department of Texas—on General Grant's staff—was promoted to the rank of general, on August 1st, and retired on account of the age limit August 7th instant. Dr. Cleary was an army surgeon in the war of secession. He is well known in Texas, and is very popular with the medical profession and all who know him. He is a delightful old fellow, a typical Irishman (and the Irish are the finest people in the world). Dr. Joseph B. Girard, U. S. A., Washington, D. C., succeeds Dr. Cleary as chief surgeon. Dr. Girard ranks as colonel. The West Texas Medical Society, at a meeting in San Antonio, August 5th, gave a "Doctors' Smoker," complimentary to the members from adjoining counties, and at a business meeting after the smoker elected General Cleary an honorary life member of the association. The garrison at Fort Sam Houston fired a salute of eleven guns in honor of General Cleary's promotion.

---

Pasteur Institute for Texas.—The last Legislature appropriated $5000 for a Pasteur Institute for Texas. It is to be an annex of the State Insane Asylum at Austin, and is to be in charge of Superintendent Worsham. The appropriation is to cover building and equipment. Indigent patients are to be admitted and treated free of charge; those able to pay will be charge a nominal fee. Heretofore, all persons bitten by rabid animals were sent to New York, St. Louis or Chicago for treatment, if able to bear the expense. The Board of Trustees of the State Insane Asylum let the contract for the building August 7th instant, and it is expected that the institution will be ready for patients by December 1st.

---

I am requested to state that Dr. M. K. Lott, in consequence of injuries received recently in a railroad wreck, and sickness resulting therefrom, will not receive any patients for treatment at his infirmary at Cameron for the present. Notice will be given when he resumes practice.

---

Prof. J. W. McLaughlin, M. D., of the Medical Department of the University of Texas, with his family, is summering in "Cool Colorado."

---

Our esteemed contemporary of the *Texas Medical News*, Dr. M. M. Smith, who is also Secretary of the State Board of Medical Examiners, is sojourning at Colorado Springs, Boulder, and Denver. Mrs. Smith and their fine boys, Matthew and Gerard, are with him. Lucky fellow! It is as much as the editor of the "Red-Back" can do to even *stay at home*, let alone going anywhere.

---

Let the good work go on, until every county in Texas organizes a medical society. Send me reports of organization for publication. Soon, to *not be* a member of one's local society will mean no insurance or other business; black sheep.

Dr. W. S. Carter, Professor of Physiology, Medical Department, University of Texas, Galveston, has been elected Dean of the Faculty, vice Dr. Allen J. Smith, resigned, and gone to the University of Pennsylvania.

---

In honor of the late Major Walter Reed, M. D., U. S. A.—A meeting was held at Bar Harbor, Maine, August 15, 1903, to confer with respect to a memorial in honor of the late Major Walter Reed, M. D., U. S. A., to whom the world is indebted for most important services in the investigation and the suppression of yellow fever. The call was signed by Daniel C. Gilman, Chairman of a Committee appointed by the American Association for the Advancement of Science; S. Weir Mitchell, Edward G. Janeway, William H. Welch, and Christian A. Herter.

